# Imaging DNA damage response by γH2AX *in vivo* predicts treatment response to Lutetium-177 radioligand therapy and suggests senescence as a therapeutically desirable outcome

**DOI:** 10.7150/thno.82101

**Published:** 2023-02-21

**Authors:** Edward O'Neill, Michael Mosley, Bart Cornelissen

**Affiliations:** 1 MRC Oxford Institute for Radiation Oncology, Department of Oncology, University of Oxford, Oxford, UK; 2 Nuclear Medicine and Molecular Imaging, University Medical Center Groningen, University of Groningen, Groningen, The Netherlands

**Keywords:** DOTATATE, γH2AX, senescence, DSBs, radiosensitivity

## Abstract

**Rationale:** An effective absorbed dose response relationship is yet to be established for Lutetium-177 based radionuclide therapies such as ^177^Lu-DOTATATE and ^177^Lu-PSMA. The inherent biological heterogeneity of neuroendocrine and prostate cancers may make the prospect of establishing cohort-based dose-response relationships unobtainable. Instead, an individual-based approach, monitoring the dose-response within each tumor could provide the necessary metric to monitor treatment efficacy.

**Methods:** We developed a dual isotope SPECT imaging strategy to monitor the change over time in the relationship between ^177^Lu-DOTATATE and ^111^In-anti-γH2AX-TAT, a modified radiolabelled antibody that allows imaging of DNA double strand breaks, in mice bearing rat pancreatic cancer xenografts. The dynamics of γH2AX foci, apoptosis and senescence following exposure to ^177^Lu-DOTATATE was further investigated *in vitro* and in *ex vivo* tumor sections.

**Results:** The change in slope of the ^111^In-anti-γH2AX-TAT to ^177^Lu signal between days 5 and 7 was found to be highly predictive of survival (r = 0.955, P < 0.0001). This pivotal timeframe was investigated further *in vitro*: clonogenic survival correlated with the number of γH2AX foci at day 6 (r = -0.995, P < 0.0005). While there was evidence of continuously low levels of apoptosis, delayed induction of senescence *in vitro* appeared to better account for the γH2AX response to ^177^Lu. The induction of senescence was further investigated by *ex vivo* analysis and corresponded with sustained retention of ^177^Lu within tumor regions.

**Conclusions:** Dual isotope SPECT imaging can provide individualized tumor dose-responses that can be used to predict lutetium-177 treatment efficacy. This bio-dosimeter metric appears to be dependent upon the extent of senescence induction and suggests an integral role that senescence plays in lutetium-177 treatment efficacy.

## Introduction

Despite impressive improvements in progression free survival in the NETTER-1 trial, peptide receptor radionuclide therapy (PRRT) with ^177^Lu-DOTATATE remains a non-curative therapy, with limited evidence of extending overall survival 1. ^177^Lu-DOTATATE remains highly effective at improving patients' quality of life 2, yet opportunities remain to improve therapeutic outcome by increasing the response rate. One aspect, increasing the administered dose of PRRT, is currently limited, given the high as safely administrable limit (AHASA) is the calculated absorbed dose of 23 Gy in the kidneys. This limit is based upon those established for external beam radiotherapy and concerns of renal toxicity from ^90^Y-DOTATATE 3. While routine dosimetry is not (yet) widely adopted in the clinic, because quantitation of ^177^Lu uptake in tissues has its own challenges, there is promising evidence of improved response rates if patients are given administered doses up to this AHASA limit 4. Although it may be tempting to advocate for ever increasing injected doses of ^177^Lu-DOTATATE, there are also valid uncertainties over long-term haematological toxicity including myeloid dysplasia and acute myeloid leukaemia 5, for which there are no known bone marrow dose-response relationships 6. Such risks need to be taken into consideration when making an informed treatment decision, which is made more difficult with the current uncertainty over the necessary therapeutic dose. This minimum therapeutic dose needed to treat the tumor has not been established for neuroendocrine tumors, as it has been for radioiodine treatment of thyroid cancer 7. To establish a dose threshold that is as low as reasonably achievable to reliably treat each patient, a relationship between administered and radiation absorbed dose and tumor response is required. It is likely that a general cohort-based dose-response may not be possible for neuroendocrine tumors due to its inherent biological heterogeneity 8. A dose-response relationship based upon SPECT-image dosimetry was found in a cohort of pancreatic neuroendocrine patients 9. However, when this same technique was applied to the more biologically diverse midgut neuroendocrine tumors, no significant radiation absorbed dose-effect correlation was observed 10, and there was a poorer dose-response correlation in pancreatic tumors with diameters less than 4 mm. Recent studies account for these differences between pancreatic and small intestine tumors by differences in tumor uptake of ^177^Lu across rounds of therapy 11, 12. These results indicate not only inherent differences in tumor uptake across patients affected by tumor morphology and diffusion, but also differences in the biological response to each round of treatment 13. Therefore, a direct metric of the biological response to ^177^Lu-DOTATATE therapy is warranted to account for these individual radiobiological differences, and establish a dose-response relationship taking into account the physical radiation dose from ^177^Lu and the (radio)biological response to that radiation dose.

One of the more cytotoxic types of DNA damage is the DNA double strand break (DSB). As part of DNA damage repair, one histone isoform, H2A.X, is phosphorylated around sites of DSBs, by kinases such as ATM, ATR, and DNA-PKcs. The S159-phosphorylated form of H2A.X is designated as γH2AX. We have previously shown that a radiolabelled modified anti-γH2AX antibody, ^111^In-anti-γH2AX-TAT, is capable of imaging the DNA double strand break damage 14-17, also following ^177^Lu-DOTATATE administration *in vivo 18*. Lethal levels of DNA damage induced by the beta-particles emitted by lutetium-177 are thought to be a significant contributor to the therapeutic effect of radionuclide therapies such as ^177^Lu-DOTATATE. Therefore, a means to monitor the DNA damage response to damage inflicted by lutetium-177 would be invaluable to assessing the repair capability of each tumor, and to assess its response. This strategy aims to measure the dose-response within each tumor, within each patient. Here, we developed a metric that was predictive of survival of mice, bearing SSTR2-expressing neuroendocrine tumor xenografts, treated with ^177^Lu-DOTATATE, through quantifying ^177^Lu uptake, and concurrent imaging DNA damage with ^111^In-anti-γH2AX-TAT. While this approach has potential if applied within the clinic to establish optimized therapeutic doses and adaptive therapy, it also provided insight into the importance of DNA damage and senescence in response to ^177^Lu-DOTATATE treatment.

## Imaging Concept

Due to the extended retention of lutetium-177 within the tumor, and prolonged low-dose rate irradiation of cancer cells, the presence of γH2AX foci may indicate either freshly formed or long-lived unresolved double stranded DNA breaks (DSBs). It is these unresolved residual γH2AX foci, existing for longer than 24 h that have been found to be predictive of clonogenic survival after external beam irradiation (EBRT), rather than the number of short-lived γH2AX foci (which are resolved within hours, representing repairable DNA damage) 19. For example, de-Colle et al. measured the speed with which yH2AX foci were resolved, and observed a high level of intrinsic radiation sensitivity in prostate cancer samples, after ex vivo irradiation with EBRT 20. The relative ability of tumors to repair DNA damage, and deal with unresolved DNA damage, would provide an essential insight into the functional radioresistance of tumors to lutetium-177 based therapies, particularly with heterogenous neuroendocrine and prostate tumors 8. A functional approach to assessing radioresistance towards EBRT has recently been demonstrated by employing an *ex vivo* RAD51 foci formation assay 21. However, unlike the single, brief EBRT exposure used in the RAD51 assay, it is challenging to distinguish non-invasively between the quickly resolved dsDNA breaks continually inflicted through lutetium-177 irradiation and residual foci at a single timepoint 22. Furthermore, γH2AX foci have been shown recently to correlate with sstr2 immunofluorescence expression *ex vivo* as a proxy for ^177^Lu-DOTATATE dose 23.

**Figure 1:**
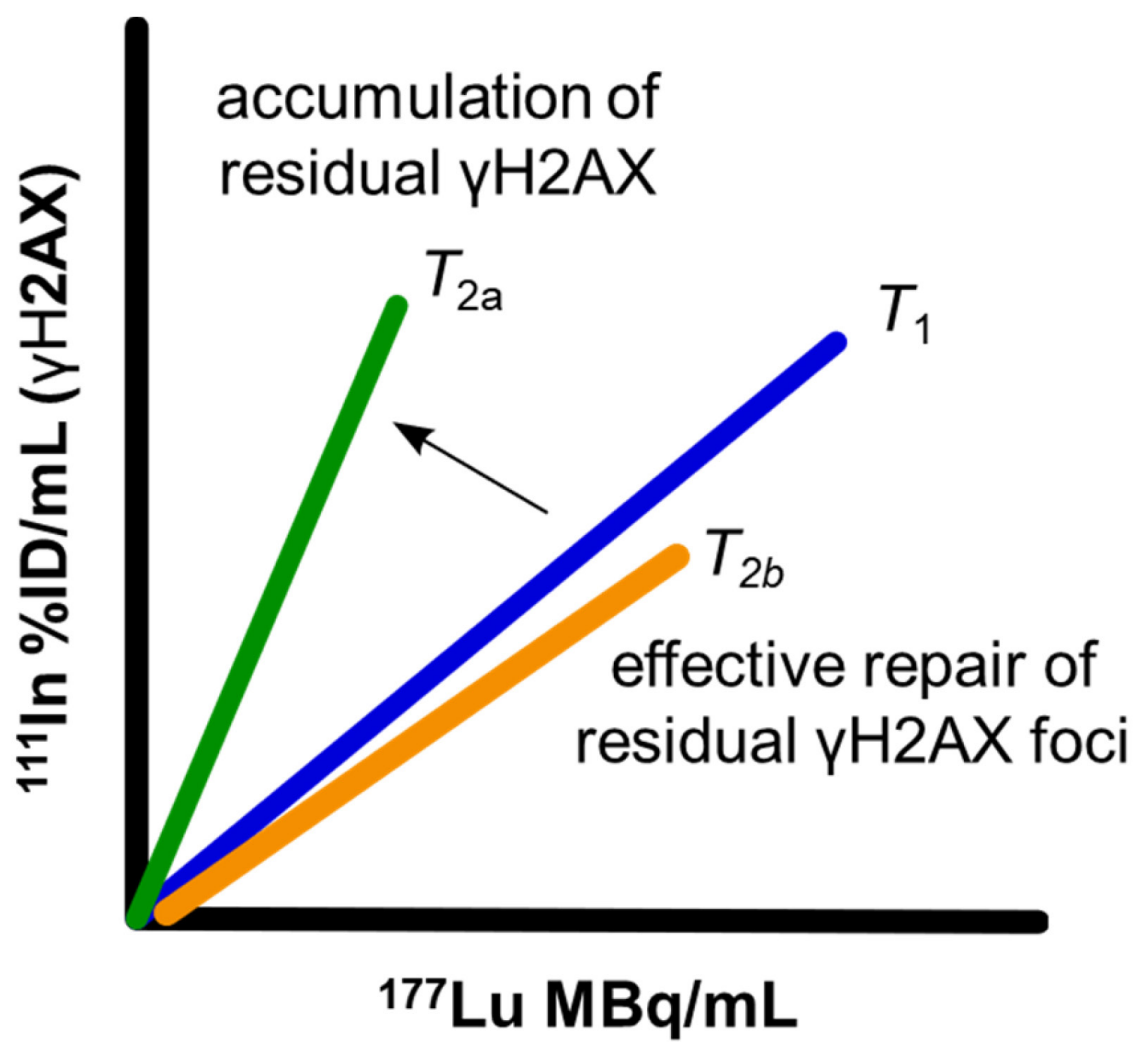
** Relative accumulation of** residual γH2AX foci can be measured through increases in the ^177^Lu to ^111^In (γH2AX) slope from an initial timepoint (*T*_1_), indicating an accumulation of residual foci (*T*_2a_) or maintenance of a slope (T_2b_) similar to slope at *T*_1_ indicating a steady-state of DNA damage repair.

To address this need, the accumulation over time of γH2AX foci, relative to lutetium-177 uptake within the tumor can be visualized and quantified using SPECT imaging with ^111^In-anti-γH2AX-TAT, and ^177^Lu. By monitoring the change of this dual-isotope relationship, the accumulation of residual foci above and beyond the steady state repair of short-lived γH2AX foci can be quantified: If the proportion of γH2AX relative to lutetium-177 decreases or remains the same over time within a tumor then this would indicate the tumor is capable of continuing to successfully resolve DSBs until lutetium-177 is eventually cleared from the tumor (Figure 1). However, if the proportion of γH2AX relative to lutetium-177 increases over time, then this would indicate accumulation of unresolved γH2AX foci above the steady-state clearance of short-lived γH2AX foci. Therefore, any increase in the slope of this dose-response could be used to measure long-lived γH2AX foci which may be indicative of therapeutic success.

Previously, this γH2AX dose-response slope has been generated by varying radiation absorbed dose irradiation *in vitro* or *ex vivo* to calculate relative radiosensitivity 20. Therefore, to measure dose-response to lutetium-177 therapy *in situ*, a dose range of ^177^Lu uptake (as a proxy for radiation absorbed dose) would be required to establish relative radiosensitivity. This spectrum of lutetium-177 uptake can be achieved by utilising the heterogenous uptake of ^177^Lu-DOTATATE across a tumor, due to differences in diffusion 24 and receptor expression 23, 25.

## Materials and Methods

Full materials and methods are presented in the supplementary material accompanying this manuscript.

### Radiolabeling

^177^Lu-DOTATATE was prepared using previously described methods 26. Carrier-free ^177^Lu was obtained from ITG (Germany), DOTATATE precursor was obtained from Cambridge Biosciences. ^177^Lu-DOTATATE was prepared to a molar activity of 50 MBq/nmol for *in vitro* use, and 86 MBq/nmol (60 MBq/μg) for *in vivo* experiments, unless otherwise stated. Radiolabeling yield was routinely >99.5%, as determined by ITLC. Immunoconjugate preparation and radiosynthesis of ^111^In-anti-γH2AX-TAT was performed using mouse monoclonal anti-γH2AX antibodies (Merck, clone JBW-301) as previously described 18. JBW-301 binds human, murine and rat γH2AX.

### *In vivo* imaging

All animal procedures were performed in accordance with the UK Animals (Scientific Procedures) Act 1986 and with local ethical committee approval. Female athymic nude mice (Envigo) were housed in IVC cages in groups of up to six per cage in an artificial day-night cycle facility with *ad libitum* access to food and water. Tumor xenografts were generated by subcutaneous injection of CA20948 cell suspensions (10^6^ cells in 100 μL PBS) in the right hind flank in two staggered batches 4 days apart (2 x 15 mice) (Scheme S1). After 14 days post-inoculation mice were randomized into treatment groups. The 1st batch of inoculated mice received ^177^Lu-DOTATATE (5-30 MBq) in 100 µL PBS by intravenous injection and after 4 days received ^111^In-anti-γH2AX-TAT (5 MBq, 5 μg, in 100 µL PBS) by intravenous injection. The second batch of mice received both injections of ^111^In-anti-γH2AX-TAT and ^177^Lu-DOTATATE on the same day and SPECT/CT images were undertaken 24 h later and subsequently every 2^nd^ day, representing days 1, 3, 5, 7, 9 and 11 post start of ^177^Lu-DOTATATE treatment. SPECT/CT images were acquired in list-mode for approximately 15 minutes using a VECTor^4^CT scanner (MILabs, Utrecht, the Netherlands) equipped with a HE-UHR-RM collimator containing pinhole apertures of 1.8 mm diameter. Tumor growth was monitored by calipers and mice were culled when they reached predefined ethical endpoints including tumors >1200 mm^3^.

### Dual isotope voxel analysis

Reconstruction of both ^177^Lu and ^111^In SPECT images were performed using a SROSEM algorithm using the same number of subsets and iterations over all imaging sessions, (^111^In: 149-189 and 223-272 keV, 4 subsets, 4 iterations, no filter; ^177^Lu: 196-216 keV, 2 subsets, 4 iterations, no filter), using 0.6 mm^3^ voxels. Reconstruction of ^177^Lu and ^111^In signals using this approach were shown previously to provide reliable measurements of either isotope, by phantom studies using solutions with mixed isotope standards 27. Reconstructed images were co-registered to CT images for attenuation correction. Tumor volumes of interest were determined by using a decay-corrected 0.5%ID/mL threshold for ^177^Lu, using PMOD v.3.38 (PMOD Technologies, Zurich, Switzerland). Linear regression of ^111^In (%ID/mL) and ^177^Lu (MBq/mL) in co-registered voxels was performed in GraphPad Prism v9 (GraphPad Software, San Diego, CA, USA). For the purpose of linear regression, the signal of ^111^In was decay corrected, whereas ^177^Lu-DOTATATE was not.

## Results

The rat pancreatic CA20948 tumor model employed here has previously been widely investigated in syngeneic rat models of PRRT therapy. Our previous investigation provided proof-of-concept of an increase in γH2AX expression and uptake of ^111^In-anti-γH2AX-TAT at day 3 post administration in xenograft tumors in mice treated with 20 MBq of ^177^Lu-DOTATATE, compared to untreated animals or isotype matched ^111^In-anti-IgG-TAT controls 27. Here, to determine whether ^111^In-anti-γH2AX-TAT imaging could be used to predict survival, a large range of doses of^ 177^Lu-DOTATATE was used (0, 5, 10, 20, 30 MBq) in 30 mice (n = 6 per treatment group, Supplemental Scheme S1).

### ^177^Lu-DOTATATE dose-response

**Figure 2:**
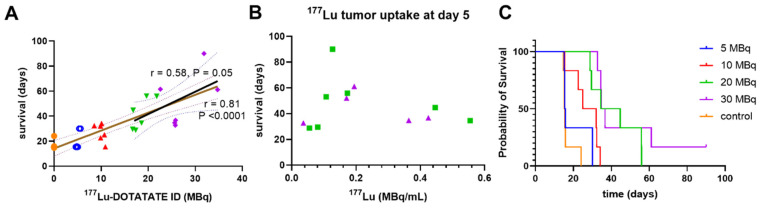
Higher injected doses of ^177^Lu-DOTATATE **(A)** correlated with survival (r = 0.81, P < 0.0001), but (**B**) retention of ^177^Lu-DOTATATE at day 5 did not correlate with survival. **(C)** Higher injected doses of ^177^Lu-DOTATATE did increase the probability of survival.

We observed a notable injected dose-response across all treatment groups with ^177^Lu-DOTATATE in this model that significantly correlated with survival (Figure 2A, Pearson r = 0.81, P < 0.0001). While this correlation was expected across a very wide dose range including very low injected doses, and using a monoclonal tumor cell-line derived xenograft, this correlation diminished markedly at therapeutically relevant higher injected doses in the 20-30 MBq range (Figure 2A, Pearson r = 0.58, P = 0.05). Similar to a clinical study in midgut neuroendocrine tumors 10, we observed no correlation between survival and mean tumor lutetium-177 uptake found for these higher-treated groups by SPECT (Figure 2B, Supplemental Figure S1). There was no statistical difference in survival between mice of the 5 MBq treatment cohort and controls (Figure 2C, Mantel-Cox, p = 0.31). Since the relatively fast growth rate of the 5 MBq treatment group tumors obscured comparison between imaging timepoints, and such a fast growth rate is not reflective of clinical experience they were excluded from further analysis.

### Measuring dose-response to lutetium-177 using ^111^In-anti-γH2AX-TAT *in vivo*

**Figure 3:**
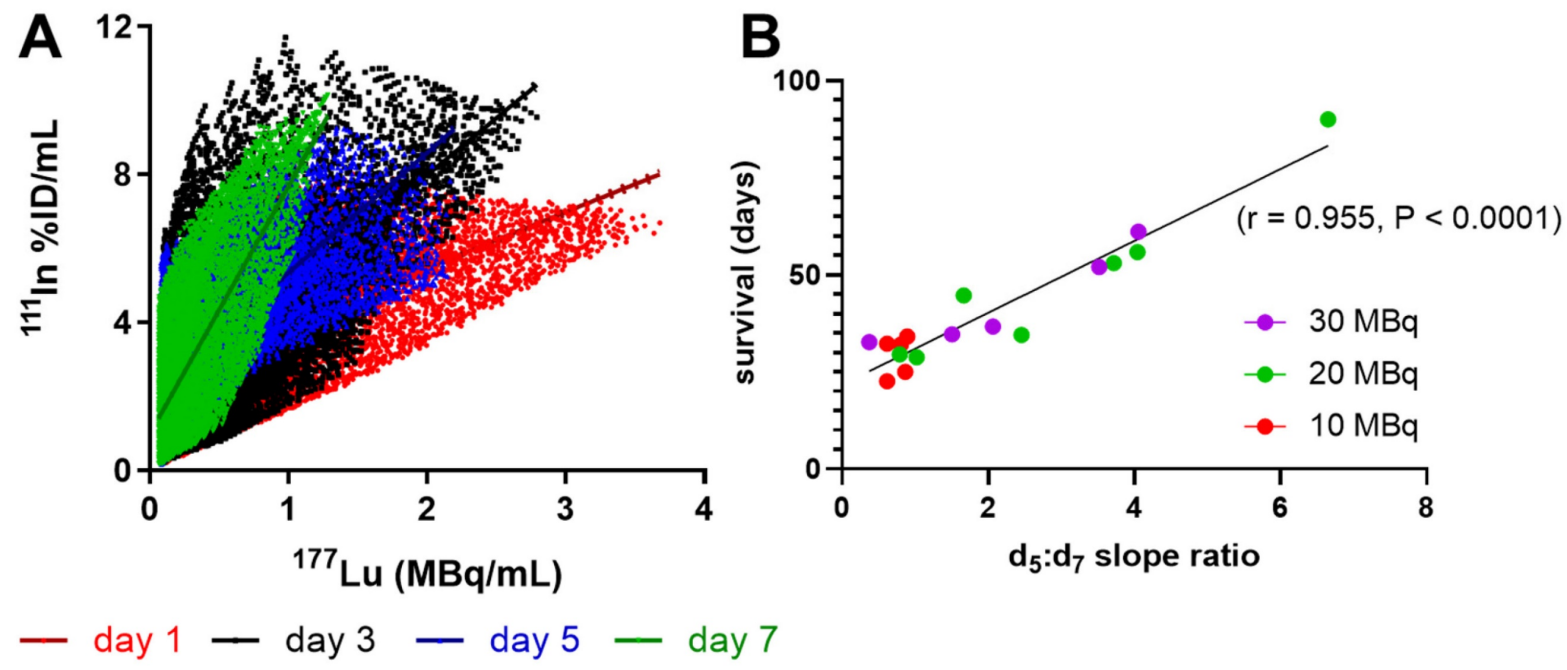
** Shift in ^111^In-anti-γH2AX-TAT to ^177^Lu-DOTATATE dose response correlates with survival. Linear regression of all** ^111^In to ^177^Lu values within each CA20948 tumor at each timepoint revealed a large shift in the slope of this trajectory between days 5 and 7. **(A)** representative mouse ^111^In to ^177^Lu trajectories imaged at days 1, 3, 5 and 7 injected with 25.8 MBq with a 37 day survival. **(B)** The ratio between slopes of the dose-response trajectories at day 5 and 7 significantly correlated with survival (Pearson r = 0.955, P < 0.0001).

A large change between days 5 and 7 was observed in the shape of the voxel-per-voxel ^111^In vs. ^177^Lu scatter-plots in mice with longer survival times (Figure 3A). Comparing the ratio of the ^111^In:^177^Lu slopes at days 5 and 7 within mice of the 10, 20 and 30 MBq treatment groups, we observed excellent correlation with survival (Figure 3B, Pearson r = 0.95, P < 0.0001). This ratio appeared to be reflective of the DNA repair capacity of the tumor with ratios >>1 correlated with greater therapeutic success while tumors with a ratio ~1 were indicative of shorter survival and therefore within the repair capacity of the tumor.

### Investigating the time course of the* in vitro* γH2AX and ^177^Lu-DOTATATE dose response

**Figure 4:**
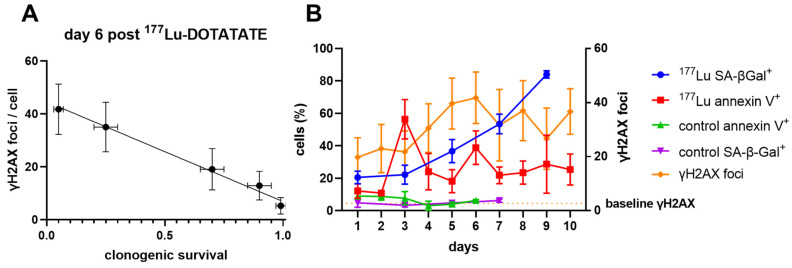
** (A)** γH2AX foci (mean ± SD) correlated inversely with clonogenic survival at day 6 (Pearson r = -0.995, P < 0.0005). Clonogenic survival values (mean ± SD) for the doses of ^177^Lu-DOTATATE determined previously 1. **(B)** Increased expression of γH2AX does not correspond to increases in apoptosis alone and sustained expression of γH2AX could reflect increased expression of senescence over time.

To understand the dynamics of γH2AX across the spectrum of ^177^Lu-induced radiation damage, we enumerated γH2AX foci by immunofluorescence in CA20948 cells up to 11 days after a brief exposure to increasing doses of ^177^Lu-DOTATATE* in vitro* (Supplemental Figure S2). Only G1 cells were analysed to minimize counting of γH2AX foci induced by replicative stress. There was a consistent linear response of γH2AX foci relative to ^177^Lu-DOTATATE up to 0.32 MBq/mL up to day 5 (Supplemental Figure S3A). The consistency of this dose response slope *in vitro* supported our observations *in vivo* and may represent the steady state DNA repair capacity of the cells. Beyond the 0.32 MBq/mL dose threshold there was a very different dose response over time with effectively no dose-response up to day 3 (Figure S4). After day 4 the slope steadily increased up to day 6 and afterwards became highly variable. Day 6 also appears to be a critical timepoint *in vitro*, as γH2AX foci across all the doses was inversely correlated with clonogenic survival (Pearson R = -0.995, P < 0.0005, Figure 4A).

**Figure 5:**
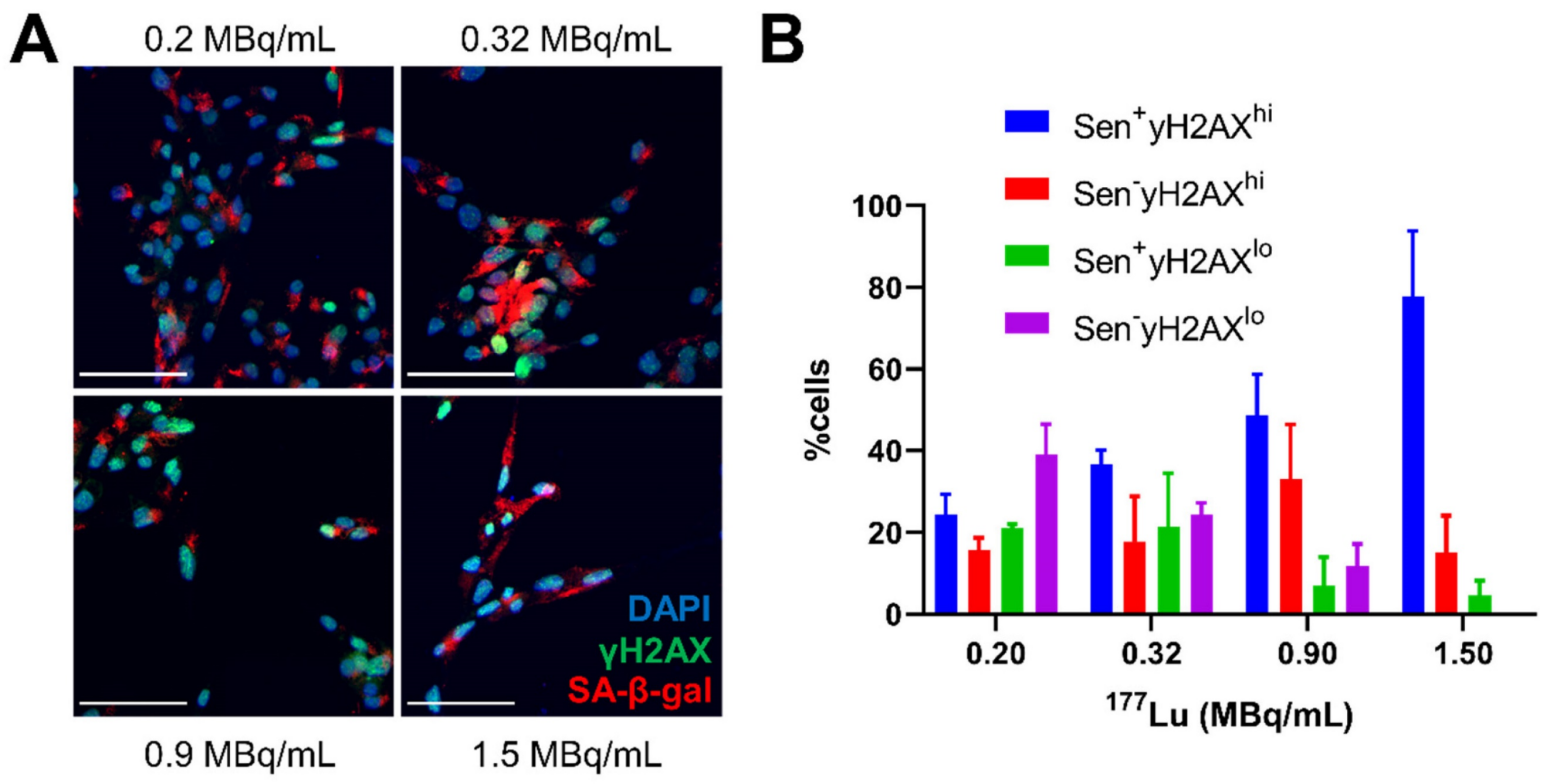
Senescence markers associated with γH2AX expression *in vitro*. (**A**) representative immunofluorescence images of CA20948 cells at day 5 post-treatment with varying concentrations ^177^Lu-DOTATATE (0.2-1.5 MBq/mL, 2 h), senescence associated β-galactosidase (red), DAPI (blue), γH2AX (green), scale bar is 100 µm. (**B**) Increasing percentage of cells with high expression of γH2AX foci with increasing ^177^Lu-DOTATATE dose concentration as well a higher proportion of these cells positive for SA-β-gal.

Since apoptosis and senescence could be contributing to the γH2AX foci expression over time, cells treated with ^177^Lu-DOTATATE (1.5 MBq/mL) were probed for apoptosis using Annexin V staining or for senescence by beta-galactosidase activity (SA-β-gal^+^) (Figure 4B). There appeared to be two distinct waves of apoptosis: at day 3 and then another at day 6. In addition, we observed a steady increase in the fraction of SA-β-gal^+^ positive cells after day 3 indicating increasing dominance of cellular senescence over time. This increased proportion of cells displaying a senescent phenotype was also dose-dependent with ^177^Lu-DOTATATE and was associated with increased expression of γH2AX (Figure 5).

### Evidence of ^177^Lu-DOTATATE induced senescence *ex vivo*

**Figure 6:**
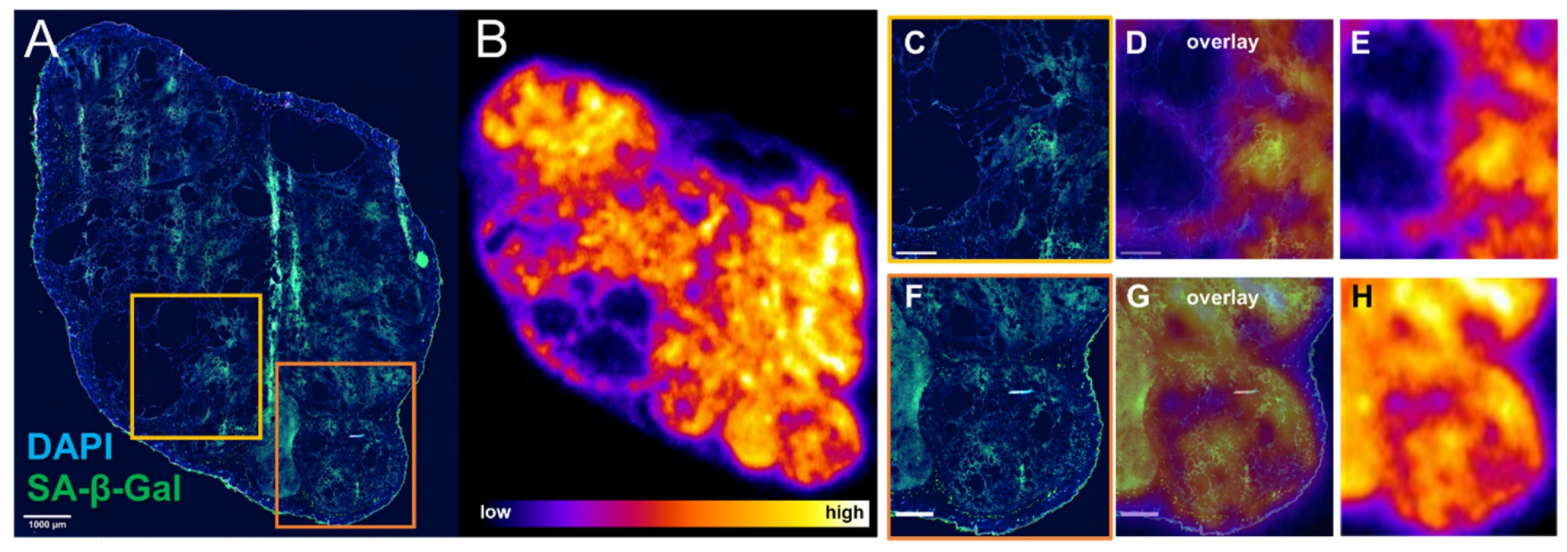
Senescence marker SA-β-gal is associated with retained expression of ^177^Lu-DOTATATE *ex vivo*. Mouse bearing CA20948 tumor was injected with 10.7 MBq ^177^Lu-DOTATATE and culled at day 11 post-injection. (**A**) Immunofluorescence of tumor tissue stained with DAPI (blue) and SA-β-gal (green) (scale bar 1 mm) was compared with (**B**) lutetium-177 autoradiography performed on an adjacent slide. (**C,F**) Regions with SA-β-gal expression were associated with higher levels of lutetium-177 (**D,E** and **G,H** scale bar 500 µm).

**Figure 7:**
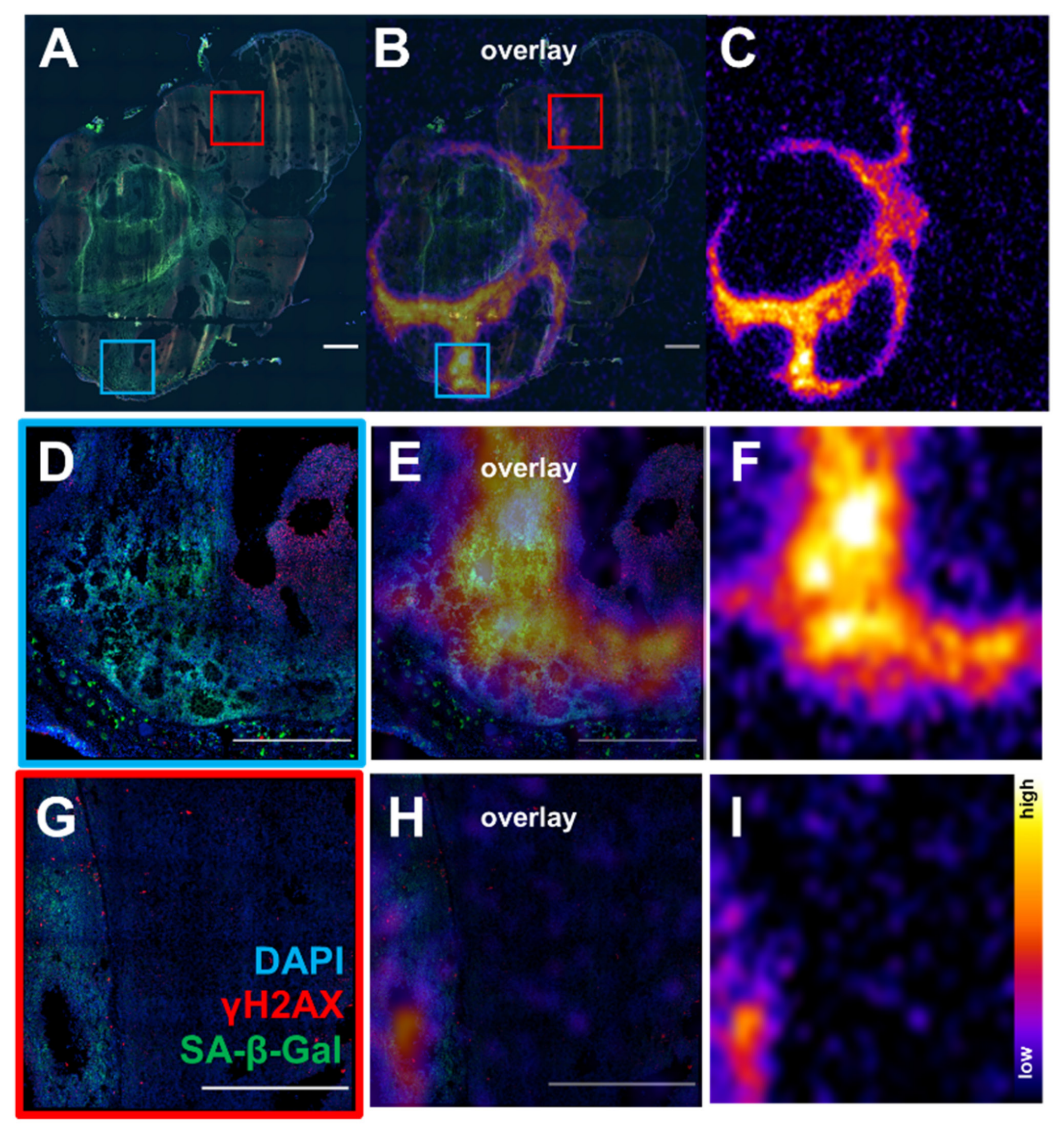
*Ex vivo* analysis of tumor from mouse treated with 16.8 MBq of ^177^Lu-DOTATATE sacrificed at day 42 post-treatment start (**A**) Immunofluorescence DAPI (blue) γH2AX (red) and SA-β-gal (green) (scale bar 1000 µm) (**C**) whole tumor ^177^Lu autoradiography from an adjacent slide and overlay (**B**). Regions with SA-β-gal expression were associated retention of lutetium-177 (**D,E,F**) and (**G,H,I**) (scale bar 500 µm). There were regions with high expression of γH2AX not associated with ^177^Lu expression and is likely due to replicative stress due to rapid tumor growth.

Since there appeared to be an association between sustained γH2AX expression and senescence in cells exposed to ^177^Lu-DOTATATE in delayed timepoints (day 5 onwards), the distribution of lutetium-177 by *ex vivo* autoradiography and SA-β-gal immunofluorescence of the tumor xenografts were investigated. Expression of SA-β-gal was associated with higher retention of ^177^Lu-DOTATATE within tumor tissue (Figure 6). These senescent tissue regions also appear capable of retaining ^177^Lu-DOTATATE for a long period, being observed at 24 days post-injection (Supplemental Figure S5), and even at 42 days post-injection (Figure 7). Even in mice therapeutically undertreated with as little as 5 MBq, we observed SA-β-gal association with lutetium-177 retaining remnant regions (Figure S6 and S7).

## Discussion

This study determined a dose-response relationship with ^177^Lu-DOTATATE in a preclinical model based not on injected or accumulated lutetium-177 activity, but rather the accumulation of DNA damage, relative to ^177^Lu. Determining a dose-response with ^177^Lu-DOTATATE, and other lutetium-177 based therapies, has been a challenge in the clinic 11. The heterogenous dose-response at therapeutically relevant doses was reflected in our study. Despite using a monoclonal cell culture derived xenograft grown in in-bred immune-compromised mice, considerable heterogeneity was seen in treatment response relative to similar injected ^177^Lu activity and ^177^Lu tumor uptake, when administering therapeutically relevant doses of ^177^Lu-DOTATATE (Figure 2). Differences in diffusion and blood vessel density between tumors and between patients is one of the challenges to establish an injected dose-response in patients treated with ^177^Lu-DOTATATE. Recent work showed diffusion weighted MRI to be an independent predictor of patient outcome 28. In the present study, this inherent heterogenous distribution of ^177^Lu-DOTATATE was used by us to monitor dose-response changes for each tumor over time.

Previous *ex vivo* analysis of residual γH2AX foci after exposure to differing doses of external beam radiation provided functional measurement of tumor radiosensitivity. Due to inherent radiobiological differences between external radiation and radionuclide therapy, any assessment of relative radiosensitivity towards^ 177^Lu-DOTATATE should not be assumed from external radiation. However, it is not possible to separately measure residual γH2AX foci from ^177^Lu-DOTATATE *in vivo* as γH2AX is constantly generated in response to ongoing lutetium-177 beta particle emissions. This study addressed this challenge by measuring the change in ^111^In-anti-γH2AX-TAT signal, as a proxy for γH2AX foci numbers, relative to varying ^177^Lu-DOTATATE doses over time, and within each tumor, to indirectly measure the accumulation of γH2AX induced by ^177^Lu-DOTATATE. While the slope of the dose-response curve was previously used as a metric for functional radiosensitivity in the *ex vivo* γH2AX assay 29. Here we used the change in this slope as a measurement of γH2AX as a metric of residual γH2AX foci. The change in ^111^In:^177^Lu slope between days 5 and 7 post injection of ^177^Lu-DOTATATE proved to be highly predictive of survival (r = 0.955, P < 0.0001, Figure 3).

This aggregate measurement of the ^177^Lu dose-response curves provides some insight into potential thresholds of the injected dose required to elicit meaningful treatment of the tumor. Mice receiving 10 MBq ^177^Lu-DOTATATE were clustered just below a slope ratio of 1, suggesting minimal accumulation of γH2AX, and therefore well within the repair capacity of the tumor against ^177^Lu-DOTATATE. However, some mice that were given higher doses of ^177^Lu-DOTATATE in 20 and 30 MBq treatment groups could also be considered within the repair capacity of the tumor as they were also clustered around 1. The extent of this shift in dose-response slopes in the remaining mice demonstrated a linear relationship with tumor growth inhibition (Figure 2A). The sustained accumulation of γH2AX appeared critical to the success of ^177^Lu-DOTATATE treatment, particularly between days 5 and 7. This sustained expression of γH2AX despite the continued physical decay of ^177^Lu-DOTATATE has also been observed within CA20948 tumors and lung NCI-H69 tumors *ex vivo 25.* Similarly, a γH2AX-luciferase reporter transfected into a lung cancer cell line showed a peak expression of γH2AX *in vitro* between day 5 and 7 in response to X-ray irradiation, and after day 5 following ^56^Fe ion exposure *in vitro* 30. Importantly, this study found an elevated signal from luciferase *in vivo* after 5 days that was persistent until at least day 12 post X-ray irradiation. The late stage γH2AX expression in response to the environmental contaminant radionuclide cesium-137 has also demonstrated a pivotal change in γH2AX dose-response between days 5 and 7 31, that was also replicated by variable dose-rate external ^137^Cs irradiator 32.

The implications of this sustained accumulation of γH2AX were interrogated further *in vitro*. The consistency of γH2AX foci generated in response to lower amounts of ^177^Lu-DOTATATE (≤ 0.32 MBq/mL) up to day 5 aligned well with the steady-state dose response relationship observed *in vivo* (Figure S3). Despite the decreasing dose-rate of ^177^Lu-DOTATATE over time, γH2AX foci instead increased over time, even after day 6 (Figure 4B). At day 6, the number of γH2AX foci per cell correlated well with decreased clonogenic survival (Figure 4A). This decrease in clonogenic survival appeared to be a result of contributions from both apoptosis and senescence. Since γH2AX expression is elevated in both apoptosis 33, and senescence 34, they may both be contributing to the increase of ^111^In-anti-γH2AX-TAT signal observed *in vivo*. However, due to the relatively slow kinetics of antibody-based imaging systems it is also likely that our imaging system is biased towards imaging of the kinetically slower target of senescent cells over the short-lived target of apoptotic cells. Furthermore, our dual-isotope imaging strategy relies upon the retention of ^177^Lu-DOTATATE, where apoptosis would result in the loss and clearance of ^177^Lu-DOTATATE.

We observed a dose-dependent increase in SA-β-Gal with ^177^Lu-DOTATATE observed *in vitro*. Within these SA-β-Gal-positive cells, an increased proportion of cells with high expression of γH2AX was found, with increasing dose of ^177^Lu-DOTATATE (Figure 5). The contribution of apoptosis in response to ^177^Lu-DOTATATE has been documented previously *in vivo* 25. In addition, apoptosis could only partially account for the sustained induction of γH2AX in the *in vitro* γH2AX-luciferase reporter study 30. The contribution of ^177^Lu-DOTATATE to tumor senescence by expression of SA-β-Gal towards lutetium-177 to our knowledge has not been documented and could account for this persistent accumulation of γH2AX, thought to be a potential trigger for senescence 35. Tumor regions with retained lutetium-177 were associated with increased expression of SA-β-Gal by *ex vivo* analysis (Figure 6). Even though mice investigated by *ex vivo* analysis in this study did experience tumor regrowth, there were regions of tumors that appeared to be successfully treated, expressing SA-β-Gal and retaining ^177^Lu, even up to 42 days post-injection (Figure 7). While this study only investigated a single administration of ^177^Lu-DOTATATE, patients will receive additional doses of ^177^Lu-DOTATATE every 42-56 days and therefore the potential impact of re-treatment of these senescent regions warrants further investigation.

## Conclusions

This study demonstrates that imaging DNA damage with ^111^In-anti-γH2AX-TAT is a suitable bio-dosimeter to predict the effectiveness of lutetium-177 based radionuclide therapy. The sustained and excessive γH2AX signal in tumors relative to retained lutetium-177 was associated with increased survival with ^177^Lu-DOTATATE therapy. If implemented in the clinic, this type of bio-dosimeter approach has the potential to guide radionuclide treatment plans according to each tumor's radiobiological response, rather than heterogenous cohort-based dose-response curves. The adoption of such (radio)biological bio-dosimeters in the clinic could provide the necessary regulatory justification to guide and optimise administration of radionuclide therapy beyond existing AHASA limits. In addition, this study highlighted the potential role of senescence following lutetium-177 treatment as a potential cause of the therapeutically successful sustained γH2AX response observed *in vivo* and therefore warrants further investigation as a potentially desirable outcome of radionuclide therapy.

## Supplementary Material

Supplementary methods and figures.
