# Supplementary material for: Imaging DNA damage response by γH2AX in vivo predicts treatment response to Lutetium-177 radioligand therapy and suggests senescence as a therapeutically desirable outcome
Source: Theranostics. 2023 Feb 21;13(4):1302–10. doi: 10.7150/thno.82101 (PMC10008745; doi:10.7150/thno.82101)

# Supporting information

### *Cell culture*

Rat pancreatic CA20948 cells were obtained from Dr Julie Nonnekens (Erasmus MC, NL), and maintained in Dulbecco's Modified Eagle Medium (DMEM), supplemented with 10% foetal bovine serum (FBS), 2 mM L-Glutamine, 100 units/mL penicillin, and 0.1 mg/mL streptomycin with 1mM sodium pyruvate. Cells were grown in a 37°C environment containing 5% $CO_2$ or in 8-well culture chambers (Ibidi) at 30,000 cells/well. After exposure of cells to $^{177}$Lu-DOTATATE (0.05 - 2.5 MBq/mL, 50 MBq/nmol) for 2 h, they were left to recover in fresh growth medium, washed with PBS and then fixed with aliquots of freshly defrosted 4% PFA. After washing, the cells were permeabilized using 0.2% triton X-100 in TBS buffer (50 mM Tris-Cl, pH 7.5. 150 mM NaCl) for 10 min at room temperature, and briefly rinsed with TBS. Non-specific binding was blocked with 2% BSA and 10% serum (donkey/goat corresponding to secondary antibody) in TBST (0.1% Tween-20/TBS) for 1 h at room temperature. Primary antibodies diluted in blocking buffer was applied directly on the slide and incubated overnight at 4°C, and then washed with TBST. Secondary antibodies were applied in blocking buffer and incubated at room temperature for an hour, washed with TBST and mounted using VECTASHEILD HardSet containing DAPI (H-1500-10, VECTOR Laboratories). Slides were then imaged using a spinning disk confocal microscope (Andor Dragonfly, Oxford Instruments).

### *Longitudinal γH2AX immunofluorescence counting*

Cells were treated as above and stained using mouse anti-γH2AX antibody (Millipore, Clone JBW-301, 1:800), and dylight-488 goat anti-mouse (Invitrogen A11034, 1:250). A 40x/1.3 oil-immersion lens was used to generate a series of tiled z-stacks that were then converted to 8 bit maximum intensity projections (MIP) in imageJ 1.51. Nuclei were masked, analysed for integrated DAPI intensity and γH2AX foci were counted within each nuclei using a defined manual intensity threshold using Cellprofiler 3.1.9. At least 250 cells were counted for each condition and timepoint.

### *Annexin V staining*

Rat pancreatic CA20948 cells were grown and treated using $^{177}$Lu-DOTATATE as above, except before fixation, cells were washed with binding buffer and stained with annexin V-Cy5 according to manufacturer's instruction (ab14150, Abcam). For each timepoint, the extent of annexin V positive cells, indicating apoptosis, was counted manually across 3 frames containing at least 100 cells each.

### **Senescence-associated β-Galactosidase staining**

Freshly fixed *in vitro* and *ex vivo* samples were stained after washing , according to the manufacturer's directions (CellEvent™ Senescence Green Detection Kit, C10851). If γH2AX staining was also required, immunofluorescence staining was also carried out as above, after the fixation step, using rabbit anti-γH2A.X primary antibody (Abcam ab11174, 1:800) and Alexa Fluor 568 donkey anti-rabbit antibody (Invitrogen A10042, 1:250). The extent of SA-β-Gal positive cells grown *in vitro* was counted manually across 3 frames containing at least 100 cells each, for each timepoint. To evaluate γH2AX SA-β-Gal co-staining, cells were manually scored to either be γH2AX$^{high}$ or γH2AX$^{low}$, and either SA-β-Gal$^{+}$ or SA-β-Gal$^{-}$ across 3 frames containing at least 100 cells within each frame.

**Autoradiography and *ex vivo* analysis**

CA20948 xenograft tumors of mice that reached their ethical endpoint were extracted following euthanasia by cervical dislocation, embedded in OCT and flash frozen using an isopropyl alcohol-dry ice bath. Tumors were cryosectioned (10 µm sections, Leica CM1850) and transferred to slides (SUPERFROST® PLUS) and stored at -80°C. To minimise the potential impact of indium-111, autoradiography for lutetium-177 was performed at least 23 days post injection of $^{111}$In-anti-γH2AX-TAT (representing at least 8 half-lives of indium-111). Phosphor screens were exposed to tumor slides for up to a week at -20°C and scanned using Cyclone Plus (PerkinElmer), and then analysed using ImageJ. Senescence and immunofluorescence staining was undertaken on slides used for autoradiography using the same techniques above for *in vitro* immunofluorescence.

***Clonogenic Survival***

CA20948 cell suspensions ($0.2\times10^{5}$ cells) were prepared using Accutase, resuspended in growth media (200 µL) and treated with either radiolabeled $^{177}$Lu-DOTATATE (0-2.5 MBq/mL, 50 MBq/nmol) and incubated at 37°C for 2 h or sham treated. An aliquot of cells for each treatment condition was plated in 6-well plates with 2 mL of growth media and incubated at 37°C in 5% $CO_2$. After 12 days, cells were washed, fixed with methanol, stained with crystal violet, and the number of colonies with more than 50 cells was counted to determine the clonogenic survival fraction.

**References**

[23] O'Neill E, Kersemans V, Allen PD, Terry SYA, Torres JB, Mosley M, et al. Imaging DNA Damage Repair In Vivo After <sup>177</sup>Lu-DOTATATE Therapy. J Nucl Med 2020;61:743-50.

**Supplementary Figures**

**Scheme S1 –** Scheme of the *in vivo* experimental protocol with two staggered cohorts designed to image across days 1, 3, 5, 7, 9 and 11

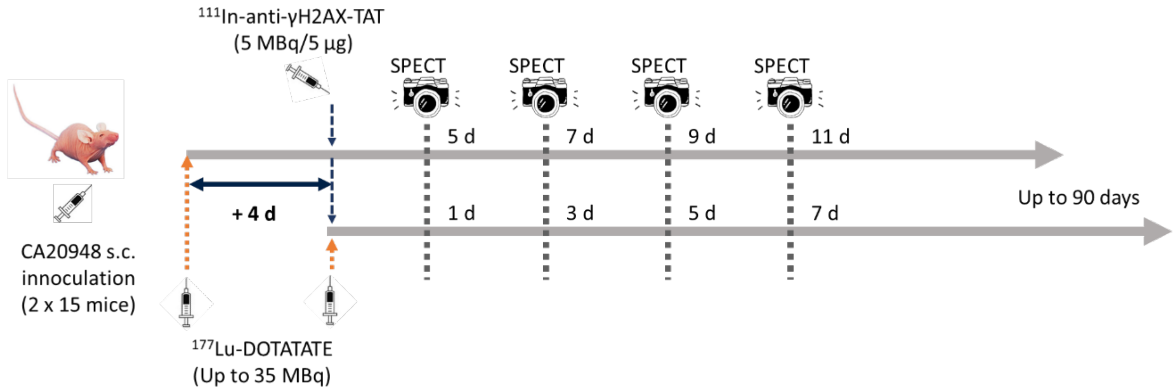

**Figure S1 –** CA20948 tumor size at start of treatment and with $^{177}$Lu-DOTATATE that underwent voxel-based analysis.

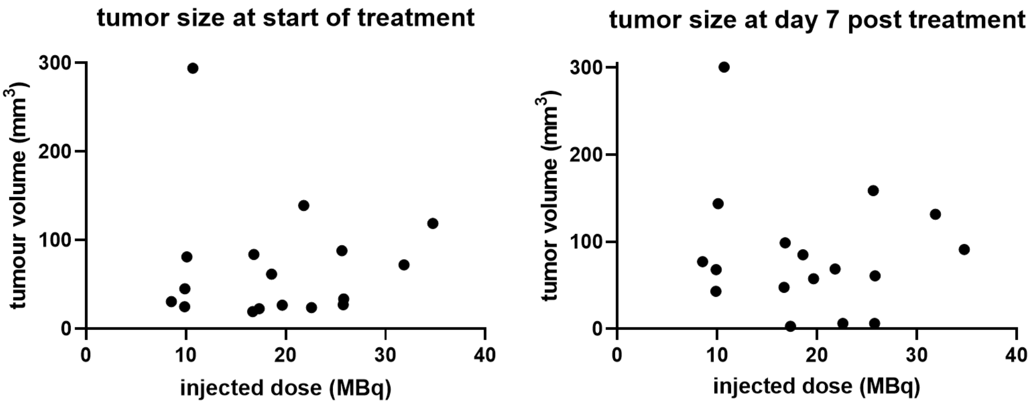

**Figure S2 – longitudinal analysis of γH2AX foci generated in response to $^{177}$Lu-DOTATATE.** CA20948 cells treated with varying doses of $^{177}$Lu-DOTATATE (0.05, 0.2, 0.32, 0.9, 1.5 MBq/mL) for 2 h, washed and left in growth media for up to day 11. Foci counting was done on immunofluorescence images automatically using Cellprofiler (>100 cells per condition, typically around 250 cells). In order to exclude cells potentially experiencing replicative stress, only G1 cells were compared gated by nuclear integrated DNA content.

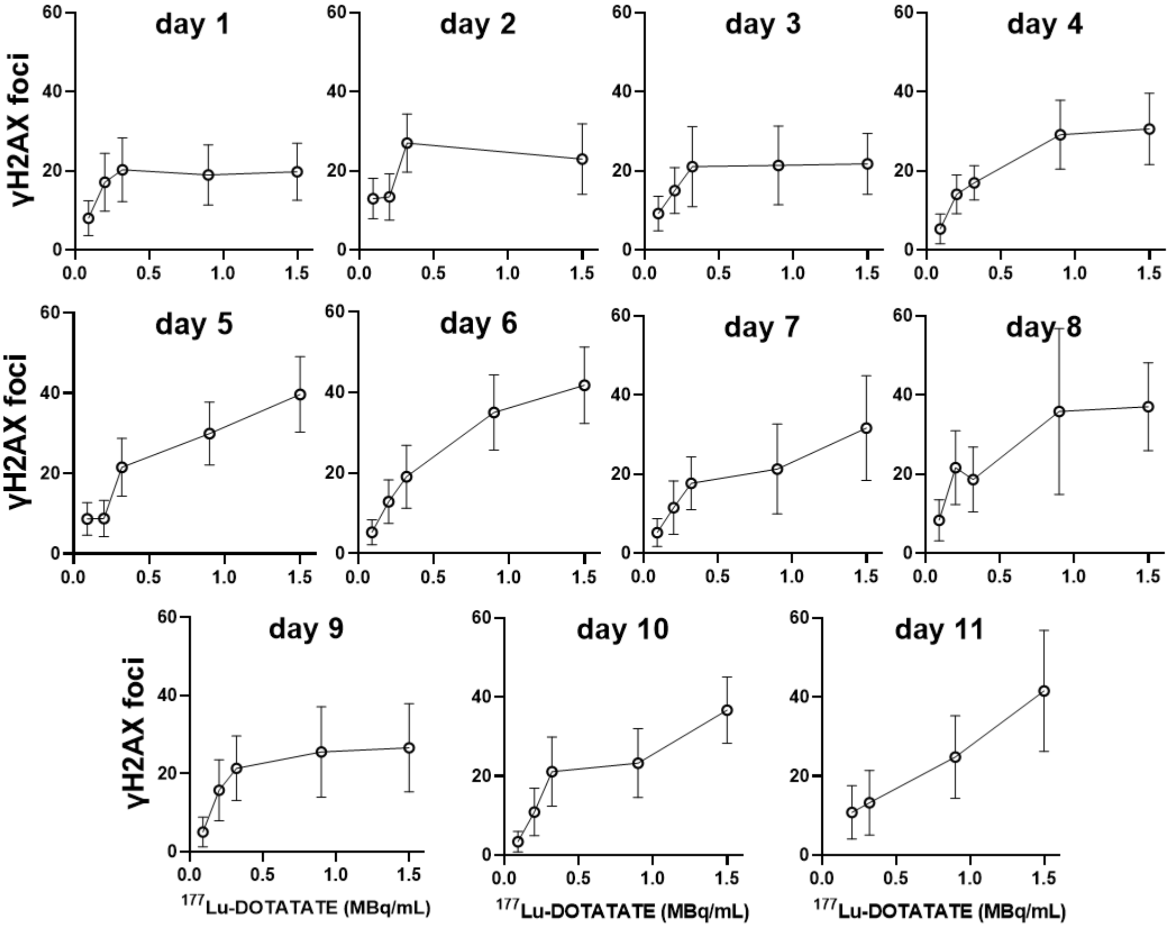

**Figure S3 – γH2AX to $^{177}$Lu-DOTATATE *in vitro* dose response up to 0.32 MBq/mL**. (**A**) There was no significant difference in the slope of the dose response curve up to day 5. (**B**) After day 5 the difference in slope became significant (*** P< 0.001)

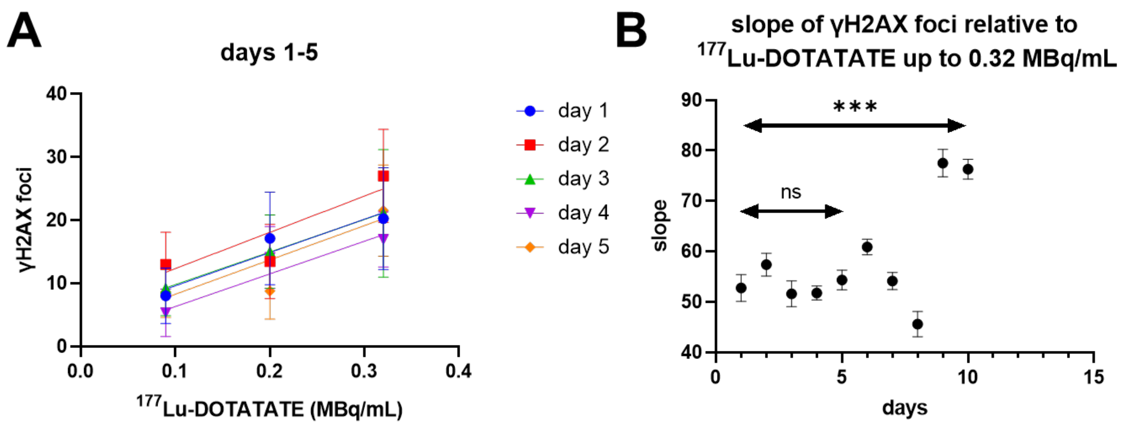

**Figure S4 – γH2AX to $^{177}$Lu-DOTATATE *in vitro* dose response beyond 0.32 MBq/mL**

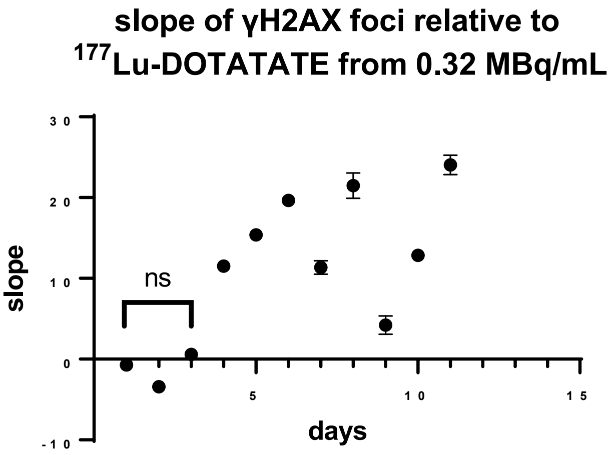

**Figure S5 -** *Ex vivo* analysis of tumor from mouse treated with 10.2 MBq of $^{177}$Lu-DOTATATE sacrificed at day 24 post-treatment start (**A**) Immunofluorescence DAPI (blue) γH2AX (red) and SA-β-gal (green) (scale bar 1000 µm) (**C**) whole tumor $^{177}$Lu autoradiography from an adjacent slide and overlay (**B**). Regions with SA-β-gal expression were associated retention of lutetium-177 (**D,G,J**) and (**E,H,K**) and (**F,I,L**) (scale bar 500 µm). At this relatively earlier timepoint compared to Figure S5, γH2AX expression is more evident alongside SA-β-gal with retained $^{177}$Lu.

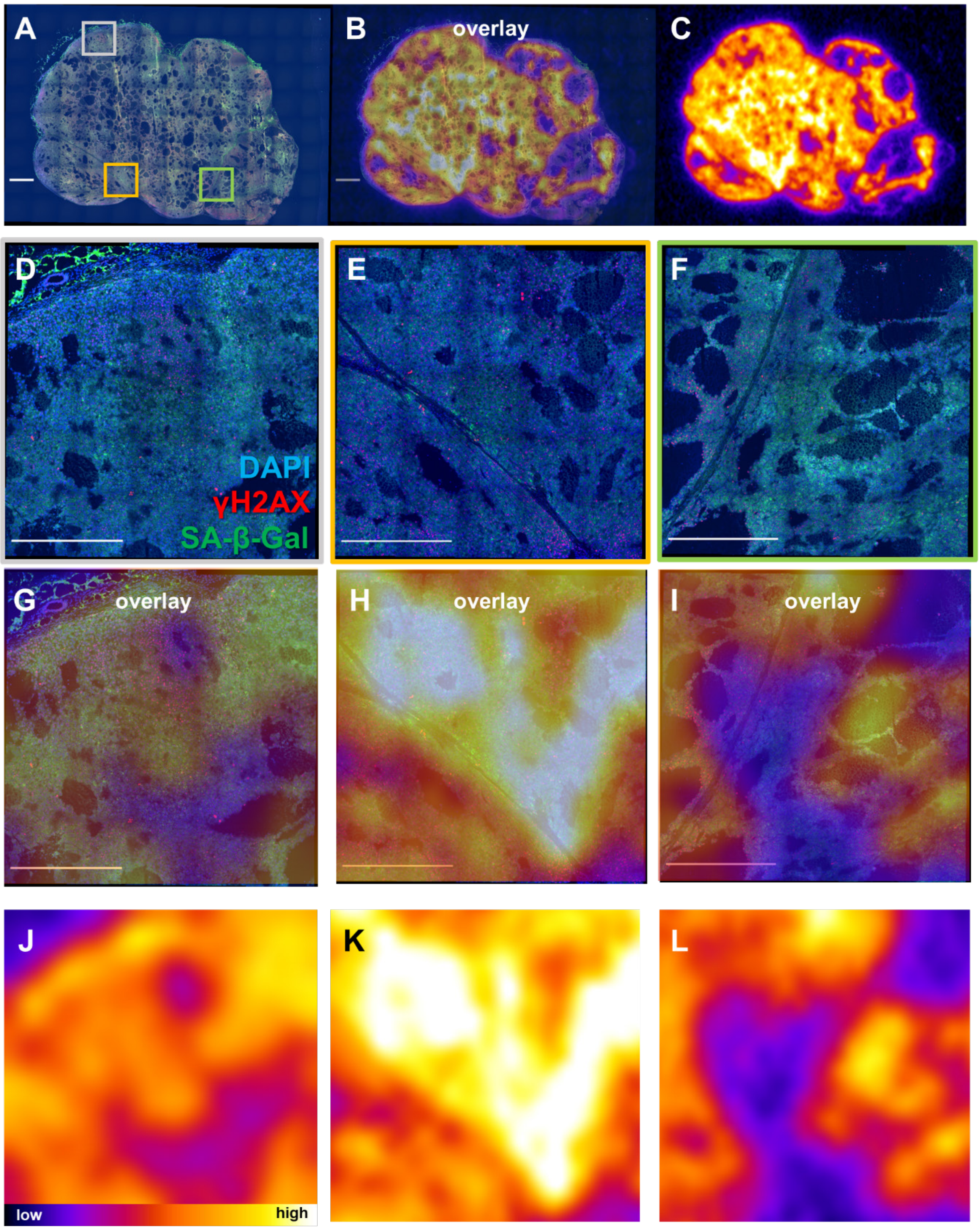

**Figure S6 -** *Ex vivo* analysis of tumor from mouse treated with 5.07 MBq of $^{177}$Lu-DOTATATE sacrificed at day 9 post-treatment start (**A**) Immunofluorescence DAPI (blue) γH2AX (red) and SA-β-gal (green) (scale bar 1000 µm) (**C**) whole tumor $^{177}$Lu autoradiography from an adjacent slide and overlay (**B**). Regions with SA-β-gal expression were associated retention of lutetium-177 (**D,E,F**) and (**G,H,I**) (scale bar 500 µm).

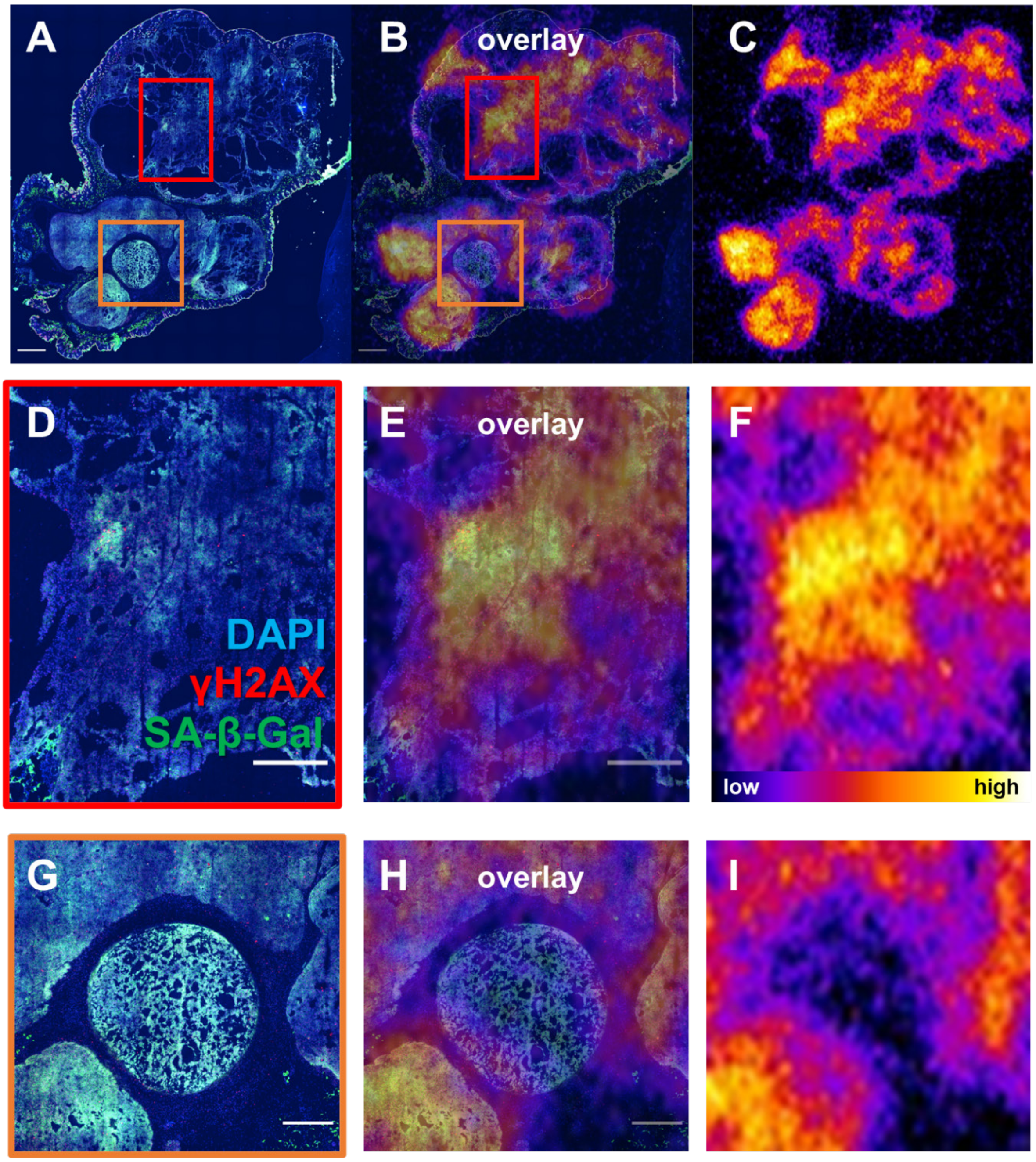

**Figure S7 -** *Ex vivo* analysis of tumor from mouse treated with 4.81 MBq of $^{177}$Lu-DOTATATE sacrificed at day 24 post-treatment start (**A**) Immunofluorescence DAPI (blue) γH2AX (red) and SA-β-gal (green) (scale bar 1000 µm) (**C**) whole tumor $^{177}$Lu autoradiography from an adjacent slide and overlay (**B**). Regions with SA-β-gal expression were associated retention of lutetium-177 (**D,E,F**) (scale bar 500 µm).

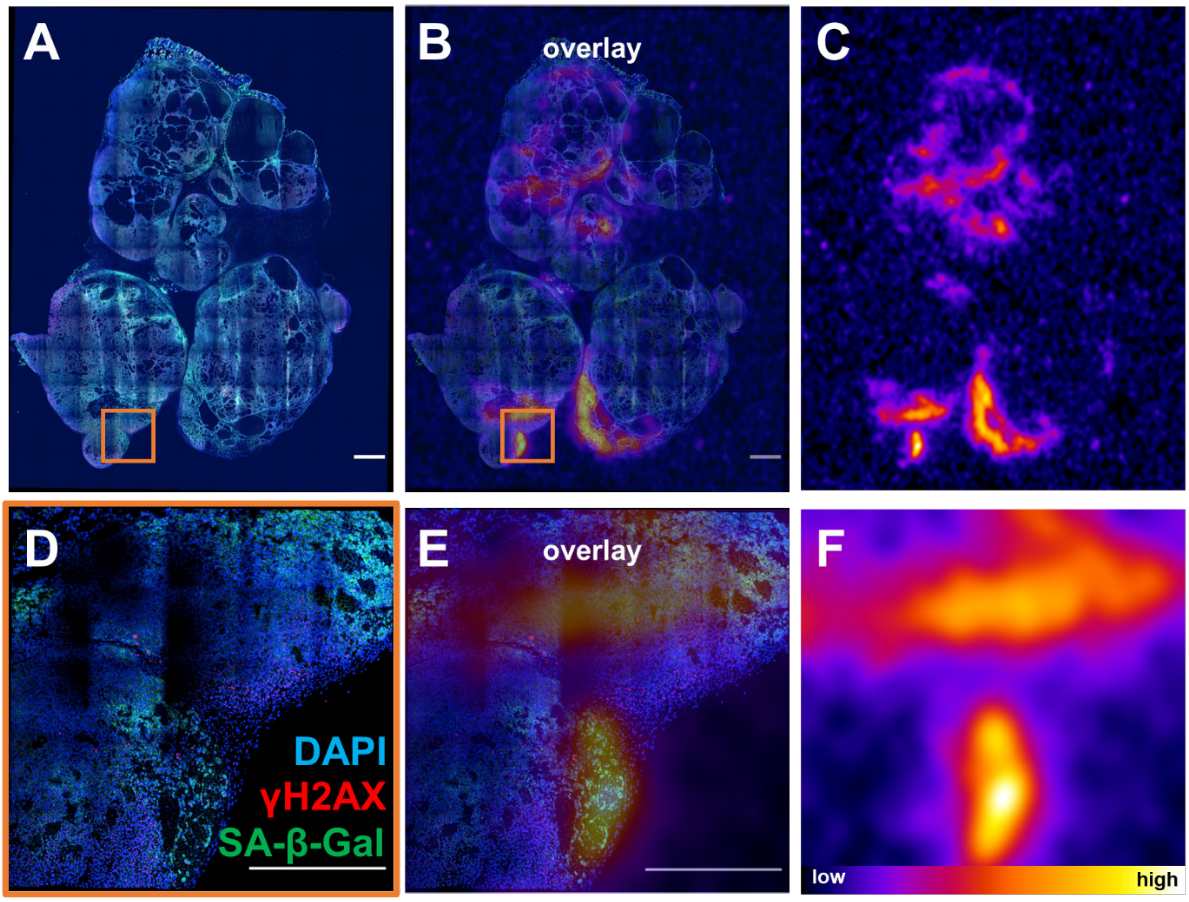

Supplement: Supplementary file 1 — Supplementary methods and figures. [file thnov13p1302s1.pdf]
